# Surface Studies of UV Irradiated Polypropylene Films Modified with Mineral Fillers Designed as Piezoelectric Materials

**DOI:** 10.3390/polym12030562

**Published:** 2020-03-04

**Authors:** Marta Chylińska, Halina Kaczmarek, Dariusz Moszyński, Bogusław Królikowski, Jolanta Kowalonek

**Affiliations:** 1 Faculty of Fine Arts, Nicolaus Copernicus University in Toruń, 30-32 Sienkiewicz St., 87-100 Toruń, Poland; mch@umk.pl; 2 Faculty of Chemistry, Nicolaus Copernicus University in Toruń, 7 Gagarin St., 87-100 Toruń, Poland; halina@umk.pl; 3 Department of Inorganic Chemical Technology and Environment Engineering, Faculty of Chemical Technology and Engineering, West Pomeranian University of Technology in Szczecin, Piastów Ave. 42, 71-065 Szczecin, Poland; dmoszynski@zut.edu.pl; 4 Institute for Engineering of Polymer Materials and Dyes, Toruń Division, 55 M. Skłodowska Curie St., 87-100 Toruń, Poland; b.krolikowski@impib.pl

**Keywords:** polypropylene, inorganic fillers, ultraviolet (UV) radiation, contact angle measurements, attenuated total reflectance-Fourier transform infrared spectroscopy (ATR-FTIR), X-ray photoelectron spectroscopy (XPS), atomic force microscopy (AFM)

## Abstract

Isotactic-polypropylene (*i*-PP) films with inorganic minerals such as Sillikolloid, perlite, or glass beads were prepared. The obtained polymeric films were subjected to an orientation process. Moreover, this paper includes results how the artificial accelerated weathering influences surface properties of the unoriented and oriented *i*-PP films with the mineral fillers. Changes in the ultraviolet (UV) treated polymeric films were studied with attenuated total reflectance-Fourier transform infrared spectroscopy (ATR-FTIR), X-ray photoelectron spectroscopy (XPS), atomic force microscopy (AFM), and by measuring contact angles. The results revealed that photooxidation of *i*-PP was more effective in the presence of the fillers and depended on the type of the filler but not on its amount. Moreover, the oriented samples experienced more effective photooxidation compared with the unoriented ones. In all studied samples the same photoproducts were detected, suggesting the same route of polymer photooxidation with and without the filler. These polymeric films were produced for potential applications in the devices in which piezoelectric effect can be used.

## 1. Introduction

Thin polymeric films are known to exhibit the piezoelectric effect, but this effect is weaker compared with that of ceramics. However, piezopolymers have advantageous characteristics that ceramics do not possess, e.g., they are flexible, less dense, cheap, and easy to produce. Piezoelectric materials based on polymers are usually classified into three groups: bulk polymers, polymer composites, and voided charged polymers [1,2].

The piezoelectric effect in bulk polymers results from their molecular structure. This group includes semi-crystalline and amorphous polymers which are dielectrics and demonstrate the ability to be polarized and to accumulate electric charges. Placing them in a strong electric field, permanent dipoles present in these materials undergo orientation showing the possibility to keep ordered electric charge in their structures. This ordered charge arises from an unordered electric charge being in this material or implemented to the material during polarization. In the case of semi-crystalline polymers, the polar crystalline phase is oriented under a strong electric field at elevated temperature. The amorphous phase, also present in such polymers, maintains the polarization of molecular dipoles in the crystalline phase. Semi-crystalline poly(vinylidene fluoride) (PVDF) is the most known piezopolymer with its piezoelectric charge constant, d_33_ about 20–28 pC/N [1,2,3,4]. The piezoelectric charge constant, d_33_, is the quantity which refers to the piezoelectric effect and is defined as the charged density generated upon applied stress. Generally, the higher d_33_ value the better piezoelectric properties of a material [1,4].

PVDF copolymers, polyamides, Parylene-C, liquid crystal polymers belong to the semi-crystalline polymers exhibiting piezoelectric properties [1,2].

Amorphous polymers have to possess molecular dipoles in their structure. Heating the amorphous polymers above the glass transition temperature, T_g_, and applying a strong electric field orients the present molecular dipoles. Afterwards, lowering the temperature in the presence of the electric field makes the molecular dipoles frozen, and the material becomes polarized. The mobility of polymer chains is greater above T_g_ and reduces below T_g_. The stiffness and brittleness are the characteristics of polymers below T_g_ in a glassy state, while elasticity and softness are typical of polymers above T_g_ in a rubbery state.

Amorphous bulk polymers encompass polyimides and polyacrylonitrile [1,2].

Combination of piezoceramics and polymers results in the formation of polymer composites in which the piezoceramics particles are dispersed in the polymer matrix. The aim of such procedure is to obtain the polymer composites based on materials whose advantageous features are used, e.g., high dielectric constant of piezoceramics and good flexibility of a polymer. The polymer does not have to be a piezopolymer, usually polydimethylsiloxane (PDMS) or poly(vinyl chloride) (PVC) are used for the preparation of the polymer composites [1,2,3,4].

Voided charged polymers, called also cellular polymers, have gas voids inserted in them. The gas molecules in these holes can be ionized after applying a strong electric field. The obtained opposite charges accumulate on the both sides of the voids producing dipoles. The voids become the trap for the induced electric charges. The piezoelectric effect appears when the charged voids are deformed. To improve the cellular structure of the polymer and consequently piezoelectric properties, inorganic microparticles are introduced to the polymer during film formation. Also, a stretching process enhances the piezoelectric effect. Cellular polypropylene (PP) and polytetrafluoroethylene (PTFE) are typical examples of piezomaterials of this group [1,2,3,4].

Piezopolymers are widely used in many different devices. They are used for production of e.g., sensors, actuators, transducers, loudspeakers, and vibration energy harvesters [1,2,3,4]. It seems that their usage will increase in the future [1,2,3,4]. The proposed polymeric piezomaterials can find applications as pressure sensors.

The *i*-polypropylene films with mineral fillers (Sillikolloid, glass beads, and perlite) exhibiting better piezoelectric properties than PVDF have been obtained lately [5,6,7]. The piezoelectric constant value d_33_ was higher for the isotactic polypropylene (*i*-PP) film with higher content of Sillikolloid or perlite (5% or 10%) than for this polymer with lower content (2.5%) of the filler. Only in the case of the samples with glass beads as the filler it was the opposite effect, i.e., the polymer with 2.5% of glass beads had the best piezoelectric properties.

The project included the creation of new materials exhibiting the piezoelectric effect. Polypropylene films with inorganic additives appeared to be such materials with piezoelectric properties much better than those of poly(vinylidene fluoride) and that is why the new piezomaterials could be attractive to industry. Moreover, the product is cheaper than commercially available PVDF.

Mechanical, morphological, thermal properties, and crystallinity of these piezomaterials have been examined previously [5,6,7]. As polymers age during the storage and usage, the resistance of the obtained materials to photoaging in accelerated artificial conditions has been tested and the results are presented in this work. The ultraviolet (UV) irradiation tests were conducted in a weathering chamber in which in the presence of oxygen atmosphere the samples were exposed to radiation similar to solar rays.

Polypropylene film undergoes photooxidative degradation when it is exposed to UV radiation. Although pure polypropylene does not contain the chomophoric groups responsible for UV rays’ absorption, this polymer is not photostable [8,9]. This behavior results from the presence of initiator residue and chromophoric groups formed during polymerization and processing at higher temperature [8]. In these places the photoreactions start by forming free radicals which then can abstract hydrogen atoms from polymeric chains and form alkyl polymeric radicals [8,9]. In *i*-PP chains there are two places from which the hydrogen atoms can be abstracted, i.e., the secondary and the tertiary carbon atoms, the latter position is privileged [10,11,12]. Alkyl radicals created in the first stage react with oxygen producing peroxides radicals which transform into hydroperoxides [8,9,10,11,12,13]. These species are not stable and fall apart creating alkoxy macroradicals and hydroxyl radicals [8,11,12,13]. Next, the polymer alkoxy radicals undergo various reactions like β-scissions, hydrogen abstractions, reactions with other macroradicals [8,10,11,13,14]. As a result of photooxidative degradation of polymers, new products bearing ketone, carboxyl, ester, perester, hydroxyl groups, etc. appear in the sample [9,10,11,12,13,14,15]. The molecular weight of the irradiated polymer decreases and low molecular weight compounds are created too [8,9,10,11,12,13,14,15].

Moreover, the alterations in the chemical structures of polymers are accompanied by the physical changes such as fragility, cracks, or color changes [8,9,10,11,12,13].

This work describes how UV radiation influences hydrophobicity, structure and morphology of the oriented and unoriented polypropylene films with inorganic fillers (Sillikolloid P87, Hoffmann Mineral GmbH, Neuburg on the Danube, Grmany), perlite, and glass beads) that differ in a chemical composition, shape, and size. Photoresistance of these materials is vital due to their potential applications outside or in the devices which are exposed to radiation. The studies of photostability of the *i*-PP films with the inorganic fillers are a novelty.

## 2. Materials and Methods

### 2.1. Materials

The polymeric films were prepared from isotactic-polypropylene (*i*-PP), Moplen HP 456J (Basell Orlen Polyolefins, Płock, Poland) and the following mineral fillers: Sillikolloid, perlite, or glass beads. Their characteristics are given in Table 1.

### 2.2. Composite Extrusion

The *i*-PP films containing 2.5, 5, or 10 wt% of the filler were prepared in extrusion processes with the usage of a co-rotating twin-screw lab extruder type Bühler BTSK 20/40D in the temperature range of 190–195 °C, with a screw speed of 300 s^−1^. Then, the obtained granulated composites were subjected to extrusion process using a single-screw lab extruder Plasti-Corder PLV 151 Brabender in the 225–245 °C temperature range, with a screw speed −75 s^−1^. The goal was to obtain a 145 mm by 0.145 mm polymer ribbon.

The obtained polymeric films had a thickness of approximately 0.10 ± 0.01 mm.

Next, a part of the obtained films was oriented (i.e., stretched 3:1). The process was carried out in two stages: at first the film was gradually heated to temperature 120–140 °C and pre-stretched, then the sample was oriented and cooled to 90–80 °C. The rollers rotated at a speed of 7–13 m/min in the first section during stretching, and at a speed of 23–30 m/min in the second section. After the orientation process, the macromolecules formed more ordered structures and the film thickness was reduced by half, except for the samples with 10% of the filler, in this case the thickness was reduced only by 10%.

### 2.3. Weathering Conditions

Artificial photoaging was performed in a Suntest XLS (xenon lamp system) device (Atlas, Linsengericht, Germany) equipped with a xenon lamp. Borosilicate glass filter cut off radiation below 290 nm, leaving ultraviolet B (UVB) (range 280–315 nm) and ultraviolet A (UVA) (range 315–400 nm) rays. Irradiation was performed in dry air without rotation and without rain simulation. Following cycles have been applied: 12 h of exposure to radiation followed by 12 h of dark period (simulation of night). 30 cycles (720 h) were used in the whole experiment. The light intensity was 350 W/m^2^, black panel temperature was 35 °C. The test conditions have been programmed.

### 2.4. Contact Angle Measurements

A drop shape analyzer (DSA) G10 goniometer (Krüss GmbH, Hamburg, Germany) was used for contact angle measurements. A drop of deionized water or diiodomethane was placed on a polymer surface and the value of contact angle was calculated on the basis of the obtained liquid drop image. About 8 drops of the liquid were placed on each sample; the deviation from the average was within ±2 deg. The measurements were carried out at room temperature.

On the basis of these measurements, surface free energies as well as polar and dispersion components were calculated by Owens-Wendt method [20] using DSA software. This method assumes that surface free energy (γ_s_) of a solid is a sum of a polar (γ_s_^p^) and dispersion component (γ_s_^d^):

γ_s_ = γ_s_^d^ + γ_s_^p^ (1)

The dispersion component describes the universal dispersion interactions which refer to London interactions. The polar component refers to polar, hydrogen, induction and acid-base interactions.

To obtain all the above-mentioned quantities, one has to conduct the measurements of contact angles, θ, of the solid with the use of two measuring liquids, polar (water) and apolar (diiodomethane). For these liquids the surface free energy, γ_l_, as well as polar, γ_l_^p^, and dispersion, γ_l_^d^, components are known. Having these data, one can calculate the surface free energy components of the solid from the Equation (2):

0.5 γ_l_ (1 + cosθ) = (γ_s_^d^ + γ_l_^d^)^0.5^ + (γ_s_^p^ + γ_l_^p^)^0.5^ (2)

However, this equation contains two unknowns (γ_s_^d^, γ_s_^p^), so two such equations give a solution.

### 2.5. X-ray Photoelectron Spectroscopy (XPS)

Prevac (Rogów, Poland) UHV system equipped with Scienta (Uppsala, Sweden) SES 2002 electron energy analyzer operating at constant transmission energy (*E*p = 50 eV) was used to acquire X-ray photoelectron spectra. As a radiation source an Al *Kα* (hν = 1486.6 eV) X-rays source was used. The binding energy (EB) calibration was done with the use of silver Ag 3d_5/2_ photoemission line EB = 368.3 eV (with reference to the Fermi level). The resolution was 1.0 eV and it was evaluated by the full-width at the half maximum (FWHM) of the Ag 3d_5/2_ peak.

The samples were attached to a molybdenum sample carrier by means of carbon conductive double-sided adhesive discs. The C 1s peak, assigned to the aliphatic carbon bindings (CH_x_) and set to 285.0 eV, was used for the correction of charging effects. The X-ray photoelectron spectroscopy (XPS) lines of other observed elements were shifted correspondingly. The reproducibility of the peak positions was ±0.1 eV.

### 2.6. Attenuated Total Reflectance-Fourier Transform Infrared Spectroscopy (ATR-FTIR)

A Vertex 70v with RT-DLaTGS Wide Range detector and ATR device (Bruker Optics GmbH, Ettlingen, Germany) with a diamond crystal (angle of incidence: 45°) was used for infrared spectra collection. The resolution was 4 cm^−1^ and the scan numbers: 64.

### 2.7. Atomic Force Microscopy (AFM)

A MultiMode NanoScope IIIa (Veeco Metrology Inc., Santa Barbara, CA, USA) atomic force microscopy (AFM) was a tool for obtaining surface images and a roughness parameter *R*_q_ (a root mean square). The images were created using a silicon tip (Veeco). The device was operating in a tapping mode in air and at room temperature. The roughness parameter was calculated for the scan area of 5 μm × 5 μm.

## 3. Results and Discussion

### 3.1. Contact Angle Measurements

Table 2 encompasses the surface free energies as well as polar and dispersion components values which were calculated on the basis of contact angle measurements with water as apolar liquid and diiodomethane as a polar liquid. It is seen from the Table that *i*-PP had a hydrophobic surface as the surface free energy and the polar component reached values 28.01 mJ/m^2^ and 2.76 mJ/m^2^, respectively. It appears to be obvious because *i*-PP contains only carbon and hydrogen atoms in its structure. The low polar component value may result from the presence of polar groups being irregularities in the polymer chains on account of polymerization, processing, or storage [8]. However, considering piezoelectric properties, these irregularities in the polymer can be advantageous because in these places the electric charges can accumulate.

The addition of the fillers to the polymer caused an increase in the surface free energy and dispersion component values, and at the same time, a slight decrease in the polar component values indicating a reduction in the sample polarity, however, the differences in these values were very small. The effect was rather related to the type of the filler not to the amount of it, which is seen in Table 2. For the samples with a given filler, regardless of its quantity, the values of the surface free energies are similar, the same applies to the values of the polar components. The decrease in the surface polarity of the polymer films with the additives, which was reflected in the lower polar component values, may indicate interactions between the macromolecules and the fillers. These additives can adsorb water molecules [6,7,16,18] and through the hydroxyl groups can interact with few polar groups present in the polymer chains as a result of polymerization or processing. Such interactions lead to the change in the positions of the polymeric hydrophilic groups towards the bulk and consequently cause a drop in the surface polarity of the films. More pronounced effect was observed for *i*-PP with glass beads and perlite as the polar components reached the lowest values for these samples suggesting stronger interactions between the polymer and these fillers. Results published earlier showed that the adhesion of a filler to a polymer was weak that is why the holes were created in the polymer film with additives, which ensued from hydrophilic nature of the filler and hydrophobic character of the polymer [6,7,21,22,23], however, some connections between the polymer and fillers were also detected [6,7].

Moreover, a sample orientation is another factor which causes a further reduction in the surface polarity of polymers, as evidenced by very low polar component values. The sample orientation is a process which makes polymer chains arranged parallel to the stretching direction [24,25,26]. The macromolecules form more ordered structure, which manifests in slightly higher crystallinity degree [6,25,26] and a considerable increase in Young’s modulus of polypropylene [6,21,22,23]. During this process macromolecules have the possibility to change their positions into energetically privileged locations due to the hydrophobic surroundings.

Furthermore, the orientation of the polymer with the inorganic fillers made the sample surfaces more hydrophobic compared with those samples without orientation, which indicated that during this process the film components had the possibility to adjust better to each other. And again, the type of the filler was more decisive factor than the amount of it, which the data in Table 2 showed. The results were similar to each other within the samples with one type of the filler. The lowest values of polar components have been noticed for the oriented *i*-PP with perlite and glass beads, which might be related to the trace amounts of water adsorbed by the mentioned fillers as the components in the film can interact with each other through hydroxyl groups. The presence of traces amount of water was detected by thermal analysis for perlite and GB, but not for Sillikolloid [5,6].

The studied films have been placed in the Suntest chamber for 1, 2, and 3 months. The 3-month-old and some of the 2-month-old samples were usually too fragile to make the contact angle measurements. Table 3 shows the results after 1 month of the UV treatment. One can notice an apparent increase in the surface free energies values, a small decrease in the dispersion components values, and a considerable increase in the polar components values. Such alterations are indicative of the formation of oxygen-containing groups on the sample surfaces owing to the photooxidation process. After one month of irradiation, one can notice that the alterations in polar components values increased more significantly for the *i*-polypropylene films with the fillers than for the neat *i*-polypropylene film, which indicated that more polar groups were created on the surfaces of the modified samples then on the unmodified *i*-PP film. The highest relative changes in the polar component values were noticed for *i*-PP with glass beads. Introduction of the fillers into the polymer film leads to the formation of voids in which oxygen molecules can be trapped and then take part in photooxidation processes, which might be an explanation for more efficient photooxidation of the polymer with the fillers. Moreover, on the filler surfaces, hydroxyl groups are present and they can catalyze photooxidation of *i*-PP too [27]. Furthermore, iron (III) oxide, a trace ingredient of all the fillers, may facilitate the polymer photooxidation, which was especially visible for the samples with glass beads. This filler had higher amount of iron oxide as compared with other additives. Fe_2_O_3_ is a semiconductor that can absorb UVA because its band gap (*E*_g_) is ~2.2 eV [28,29] in contrast to silicon dioxide and aluminum oxide which are dielectrics with much larger band gaps [28]. That is why it is possible that iron oxide can contribute to the polypropylene photooxidation. This oxide can act as a photocatalyst.

In literature, one can find some publications considering studies of irradiated PP and PE with mineral additives. Qi et al. demonstrated that the shape of TiO_2_ nanofiller influences the PP photostability. The nanorods and the spherical nanoparticles of TiO_2_ were covered with silica and then calcined. The results showed that the silica-coated and calcined TiO_2_ nanorods showed much better photostabilizing effect on PP than the silica-coated and calcined TiO_2_ spherical nanoparticles, which was explained by a larger effective shielding area and better distribution of the nanorods in the PP matrix [30]. Liauw et al. showed that gel silica hampered photooxidation of linear low density polyethylene but precipitated silica facilitated photooxidation of this polymer [31]. Mailhot B. et al. found that montmorillonite accelerated PP photooxidation [32]. Li et al. demonstrated that photooxidation and chain scission of PP was much faster in the presence of SiO_2_ because hydroxyl groups on the silica surface could catalyze PP photooxidative degradation [27]. The mentioned experiments have shown that the results are not obvious and depend on sample preparations, the type of the additives and interactions between the film components. In our experiment, the fillers used had completely different shapes, slightly different composition and size, which could affect the *i*-PP photooxidation owing to their dispersion in the polymer film and the contact area between the polymer and the additives.

Furthermore, as one can see in Table 2 and Table 3, the orientation process affected the photooxidation of polypropylene films with and without the additives. The relative changes in polar component values of the UV treated films were bigger for the oriented samples than for the unoriented ones, in general, which meant that the oriented films were more susceptible to the formation of polar groups under UV rays compared with the unoriented ones. Previously, examining the oriented *i*-PP films with the additives in terms of piezoelectric properties, the results for the oriented samples could not be achieved or the obtained results of the piezoelectric properties were much worse compared with those for the unoriented specimens [6]. The orientation process is usually applied for enhancing the piezoelectric properties by creating voids, however, in this case it was a failure. It is likely that the forces acting on the polymer during stretching caused too much deformations of voids and even cracks of the polymer chains, which resulted in macroradicals creation in these sites. Subsequently, the macroradicals formed could react with oxygen molecules and produce new functional groups which were responsible for enhanced polymer susceptibility to photodegradation [8]. It was suggested that the prime products forming during drawing were the polypropylene hydroperoxides, mainly tertiary hydroperoxides, which are not resistant to UV action and break down into radicals and these species, in turn, take part in propagation reactions of polypropylene leading to the polymer photooxidation [33,34]. Finally, the surfaces of the UV treated polymeric films became enriched in oxidized groups enhancing surface polarity of the UV treated films.

Moreover, the presence of the additives in the oriented polymeric films also promoted the photooxidation. The most significant relative increase in the polar component values compared with these values for the untreated specimens was noticed for *i*-polypropylene films with glass beads indicating the most efficient oxidation process in these specimens. The explanation of photooxidation reactions in the oriented *i*-PP films with the fillers is more complicated because the influence of two factors is imposed, i.e., film orientation and the presence of additives. In addition to specific textures of the oriented samples and possible creation of macroradicals as a results of the cleavage of macromolecules during stretching, which promotes more effective photooxidation, there are also the filler particles which are able to make photooxidation of *i*-PP film more efficient probably due to the presence of hydroxyl groups and iron oxide acting as photocatalysts. Furthermore, both the addition of the fillers and film orientation result in the creation of voids in the materials, which is related to the increase in a free volume in the polymer films. Consequently, the free volume in the polymer makes the molecules, free radicals and macroradicals movements easier, which means that photooxidation process can be more effective as well.

Only in the case of the irradiated unoriented *i*-PP films with Sillikolloid the polar component values were higher when compared with these values for the irradiated unoriented polymeric films with that filler, which suggested that these stretched films were stabilized by the ordered structure. In literature, one can find the results describing how UVA or ultraviolet C (range 200–280 nm) radiation affected photooxidation of PP drawn and undrawn multifilament yarns [25]. The authors found that the drawn filaments had a longer induction period than the undrawn ones owing to the higher content of crystalline phase and greater ordering of macromolecules in the drawn filaments, which had a stabilizing effect on the polymer photooxidation [25]. In γ-treated *i*-PP samples with different draw ratios, the processes such as crosslinking or oxidation occurred with lower efficiency with increasing draw ratio due to the increase in the content of the crystalline phase and density, which induced a drop in the free volume and radical mobility [26].

As can be seen, the type of the filler and film orientation affect the photooxidative degradation of *i*-PP films. This process was more efficient in the presence of all the fillers. The oriented films with perlite and glass were more susceptible to photooxidation than the oriented films with Sillikolloid.

### 3.2. Surface Composition Analysis

The elemental analysis of the surfaces of all studied samples carried out by means of X-ray Photoelectron Spectroscopy indicates that they contain carbon atoms with a small concentration of oxygen atoms (Table 4). The concentration of oxygen atoms at the surfaces reaches only about 2% at., which is a typical value of hydrocarbon polymers after polymerization and processing. The XPS analysis indicates that there are no mineral additives on the film surfaces before irradiation (except some Si contamination for the neat *i*-PP sample). It may indicate that the mineral compounds are tightly covered by polypropylene layer. Considering an information depth of XPS signal for C 1s spectrum, the thickness of this layer is not smaller than approximately 3 nm.

Orientation process of the *i*-PP films resulted in a drop in the concentration of oxygen atoms to about 1% at., regardless of the presence of the fillers, which suggested that functional groups from the polymer were directed inside the samples. During this process the polar groups took privileged positions due to hydrophobic surroundings and in the case of the polymeric films with the fillers some interactions between the polymer and the filler could occur.

**Figure 1:**
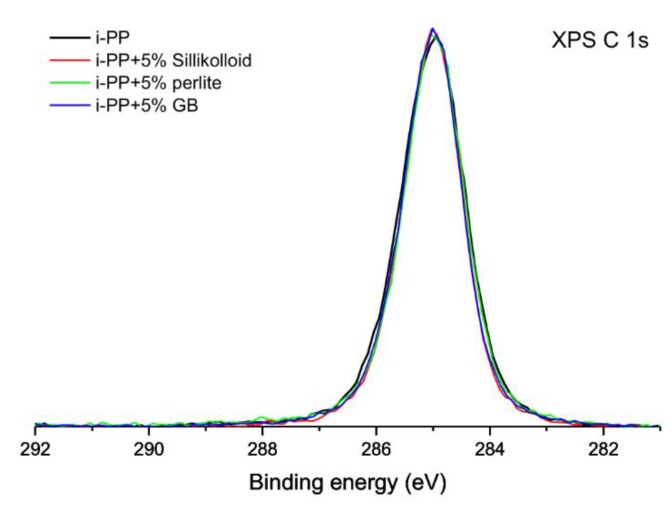
High-resolution X-ray photoelectron spectra of the unoriented isotactic polypropylene (*i*-PP) film and its films with 5% of the fillers in the energy range of the C 1s signal.

In Figure 1 the superimposed high-resolution XPS C 1s spectra of the unoriented neat *i*-PP films and the polymeric films with 5% of the fillers are shown. The C 1s spectra of the samples are typical of hydrocarbon polymers, symmetrical and with low value of FWHM (1.2 eV). The binding energy of the center of gravity of each XPS peak was set at 285.0 eV during the calibration procedure. However, it is worth noting that the peaks acquired for all considered samples overlaps perfectly. The matching XPS spectra indicate that the chemical composition of the surfaces of these samples is virtually identical. The XPS spectra obtained for the oriented materials were analyzed in the same way (spectra not shown here) and they are also identical to these shown in Figure 1.

UV radiation caused a significant increase in the concentration of oxygen atoms at the surface region of all the studied samples. After UV treatment, in most samples the oxygen content reached value of about 5% at. (Table 4). In the unirradiated samples, the oxygen content was very low about 1–2% at. This increase may result from the incorporation of oxygen atoms in the surface layers owing to oxidation processes. It can also correspond with the presence of silicon (in form of SiO_2_) observed for some samples probably due to etching process during of which a thin surface layer is removed and deeper layers become uncovered. A larger relative increase in concentration of oxygen atoms at the surface region was noticed for the oriented samples with the fillers. The presence of the fillers and the stretching process facilitated oxidation of *i*-PP. The mineral fillers were added to the polymer film to produce cellular polymeric films with enhanced piezoelectric properties. For the same reason, stretching of the film was conducted and during this operation the macromolecules are straightened and lengthened and the voids are deformed. A susceptibility of the oriented *i*-PP films with the fillers to photooxidation may result from the presence of hydroperoxides groups formed during stretching when some bonds in macromolecules become destroyed and macroradicals produced react with oxygen [33,34]. Moreover, a polymer with a cellular structure could accumulate more oxygen molecules which could react with the macroradicals and accelerate oxidation. Also hydroxyl groups and iron oxide present in the mineral fillers may act as photocatalysts.

**Figure 2:**
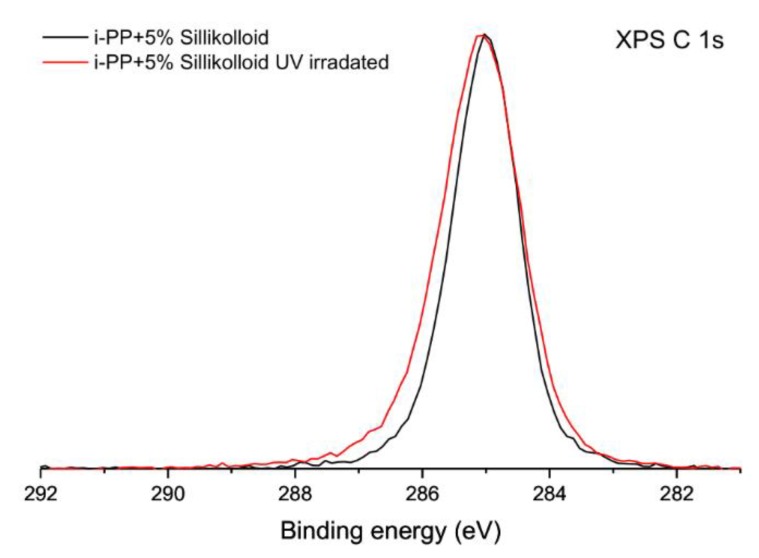
High-resolution X-ray photoelectron spectra of the unoriented *i*-PP + 5% of Sillikolloid in the energy range of the C 1s signal, black line—before ultraviolet (UV) treatment, red line—after one-month UV irradiation.

**Figure 3:**
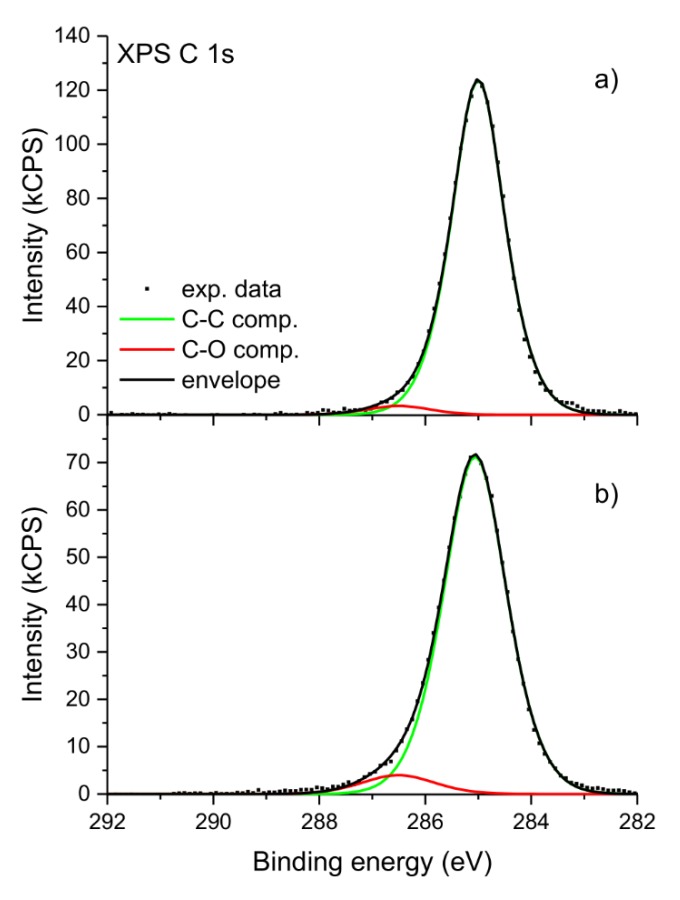
High-resolution X-ray photoelectron spectra of the unoriented *i*-PP + 5% of Sillikolloid before (**a**) and after (**b**) one-month UV irradiation.

After irradiation the XPS C 1s spectra obtained for all samples changes only slightly. An example of the sample of the unoriented *i*-PP with Sillikolloid is shown in Figure 2. The spectrum before irradiation was used as a reference. The maximum of the peak is generally placed at the identical binding energy. The envelope of the peak is less symmetrical than the one acquired for the sample before irradiation. X-ray photoelectron spectra with a deconvolution of C 1s peak for the unoriented *i*-PP with Sillikolloid is shown for the sample before irradiation (Figure 3a) and after UV irradiation (Figure 3b). Only two components were applied in the deconvolution procedure: C–C component located at the binding energy of 285.0 eV is associated with all C–C and C–H bindings present in the polymer structure and C–O component located at the binding energy of 286.4 eV. The latter position corresponds with a component usually located in the range of 286–287 eV, which is ascribed to the presence of C–O linkages in the polymer [35,36,37,38]. The relative content of carbon atoms in C–C and C–O components was evaluated for both samples. Before irradiation C–O component accounts for about 2% of all carbon atoms and after irradiation it increases to about 5% of all carbon atoms. These values are in line with the total concentration of oxygen determined by XPS analysis.

The XPS results confirmed previous findings from analysis of the surface free energy and its components.

### 3.3. ATR-FTIR Spectroscopy Results

**Figure 4:**
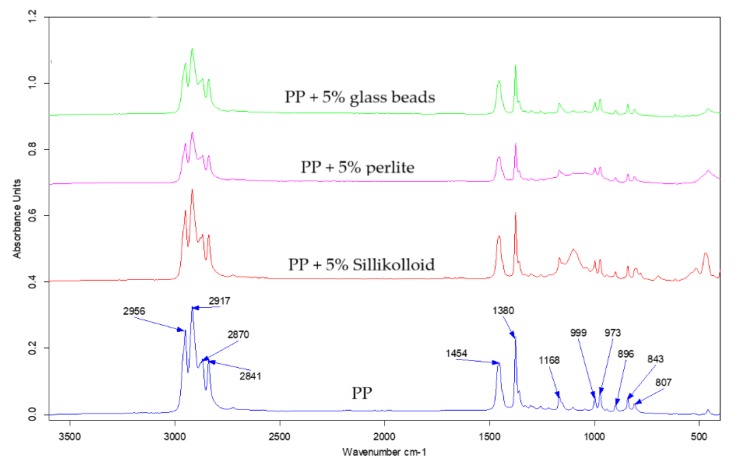
Attenuated total reflectance-Fourier transform infrared spectroscopy (ATR-FTIR) spectra of the unoriented *i*-PP (**blue**), *i*-PP +5% of Sillikolloid (**red**), *i*-PP + 5% of perlite (**pink**), and *i*- PP + 5% of glass beads (**green**).

The infrared analysis is a useful tool for identifying functional groups at the surface. The depth of gathering information is about a few micrometers, which means that the sampling depth of this technique is greater as compared with XPS and contact angle methods where the sampling depth is about a few nanometers [39,40]. The usage of infrared spectroscopy let us investigate the structure of the prepared *i*-PP films. The examples of the ATR-FTIR spectra of the *i*-PP films are presented in Figure 4. These spectra are typical of PP and one could distinguish only bands assigned to the C–H or C–C bond vibrations [41]. A detailed description and the assignment of the bands to appropriate bonds vibrations in PP was carried out in earlier papers [42,43]. However, the spectra of the *i*-PP films with various fillers differ from each other in the range of 1000–1200 cm^−1^. A clear absorption band with a maximum at 1100 cm^−1^ was visible only in the spectrum of the *i*-PP film with Sillikolloid and this band could be assigned to Si–O bond vibrations [41]. In this region of the spectrum of the polymeric film with perlite, one can observe a broad band of low intensity. The spectrum of the neat *i*-PP film was very similar to that of the *i*-PP with glass beads, both spectra had no band in this region. The ATR-FTIR spectra of the oriented *i*-PP films were very similar to these of the unoriented samples.

**Figure 5:**
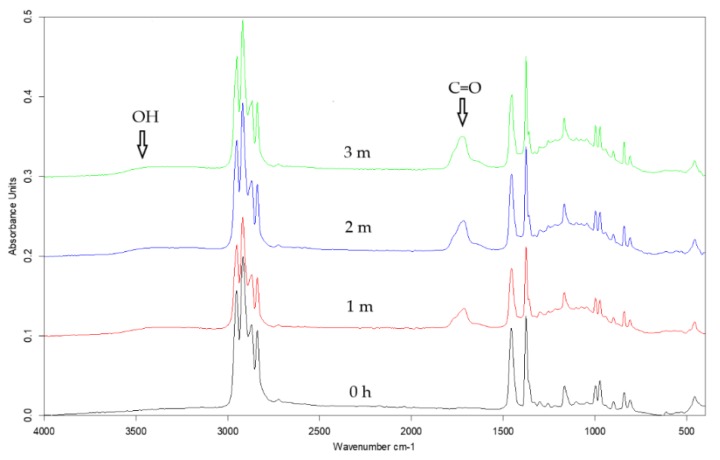
ATR-FTIR spectra of the unoriented *i*-PP + 5% of glass beads before UV treatment (**black line**), after one-month (**red line**), two-month (**blue line**), and three-month irradiation (**green line**).

Irradiation in the climatic chamber brought about the appearance of new absorption bands in the ranges of 3050–3600 cm^−1^, 1600–1800 cm^−1^, and 1000–1200 cm^−1^ in the IR spectra of the studied polymeric films (Figure 5). The first band corresponds to the vibrations of hydroxyl/hydroperoxides bonds, the second band has been assigned to the stretching vibration of C=O bond, and the third band was related to the vibrations of C–O bond [41]. The broad band in the region of stretching hydroxyl/hydroperoxides bonds vibrations indicates the existence of hydrogen bonded OH/OOH groups present in alcohols and carboxylic acids, which are the product of photooxidation of the polymer. [12,14,41,44]. In the carbonyl band, one can distinguish a maximum at about 1718 cm^−1^ and shoulders at 1770 and 1645 cm^−1^. The position of the main maximum indicates the presence of ketone groups, the shoulder at higher wavenumbers suggests the existence of peroxyesters, peroxyacids, or lactones [10,12,15,44,45]. The shoulder at 1645 cm^−1^ indicates the presence of unsaturated polymer chain ends [8,26,41,44,46]. These oxygen-containing products were created during the photooxidation of the polymer, the unsaturated groups could be formed as a result of Norrish type II reactions [8,41,44]. This type of the reaction requires space which is created by increasing the free volume of the polymer after adding the fillers and after orientation. The intensity of these bands increased with UV irradiation time, however, a quantitative analysis revealed only slight differences between the samples. The third region attributed to stretching C–O bond vibrations is indicative of the presence of oxidized products, such as alcohols, acids, ethers, esters, and lactones [10,41,44].

The photoproducts detected by infrared and XPS analysis are the same for the neat *i*-PP film and *i*-PP films with the fillers, suggesting the same mechanism of *i*-polypropylene photooxidation in the presence of the fillers and without them.

The detected in the infrared spectra the oxygen-containing products were responsible for the increase in surface hydrophilicity of the studied samples. The ATR-FTIR results confirmed previous findings from contact angle measurements and XPS.

### 3.4. AFM Results

**Figure 6:**
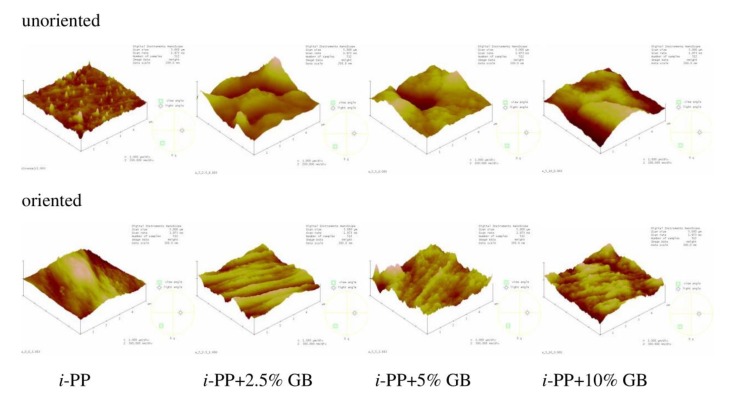
Atomic force microscopy (AFM) images of the unoriented (**top**) and oriented (**bottom**) neat *i*-PP film and *i*-PP films with different content of glass beads (GB). Scan area: 5 μm × 5 μm, z-scale for the unoriented films: 200 nm, for the oriented films: 300 nm.

**Figure 7:**
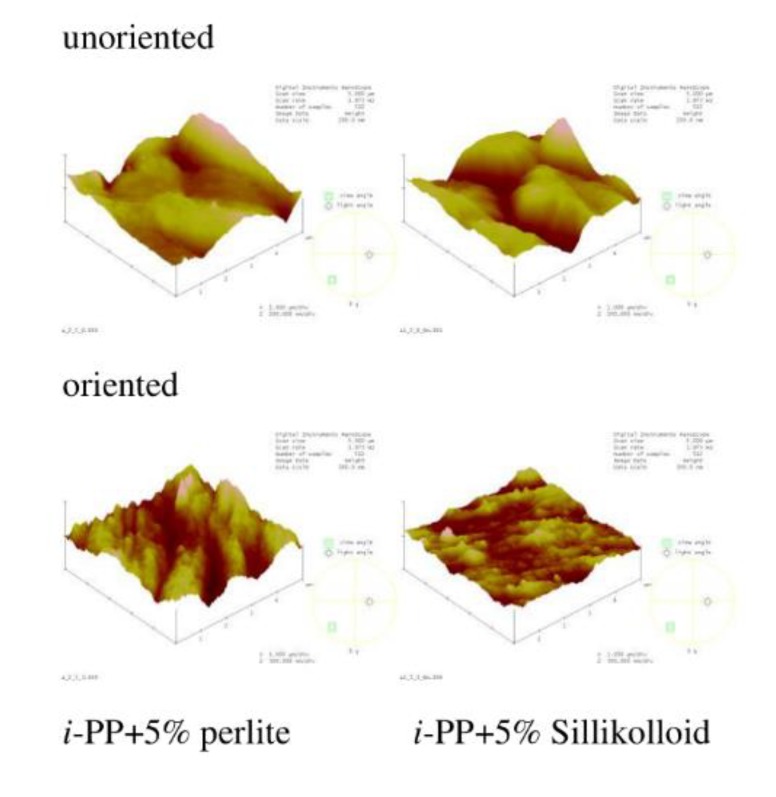
AFM images of the unoriented (**top**) and oriented (**bottom**) *i*-PP films with 5% of perlite and Sillikolloid. Scan area: 5 μm × 5 μm, z-scale for the unoriented films: 200 nm, for the oriented films: 300 nm.

AFM images and the roughness parameter (*R*_q_) were acquired to analyze film morphology. Figure 6 shows the chosen images of *i*-PP film and the polymeric films with different content of glass beads. Figure 7 presents the AFM images of polymeric films with 5% of Sillikolloid and perlite. The surface of the *i*-PP film was different from those of other films, one can notice many high and narrow cone-like structures distributed quite evenly on the surface. The films with the fillers had morphology similar to each other and the morphology was independent of the type and the amount of the filler. These surfaces were well-developed and strongly undulating with hills and valleys. Table 5 contains the roughness parameter values. It is seen that that these values are high owing to the method of obtaining films, i.e., extrusion process, and the presence of the fillers. The *R*_q_ values for the films with the fillers are clearly higher as compared with that for the neat *i*-PP film. The addition of the filler particles to the *i*-PP film caused the increased in *R*_q_ values about threefold.

The surfaces of the oriented samples differed greatly from the unoriented ones, which is seen in the Figure 6. The architecture of the oriented samples is more ordered and one can notice microfibers arranged in parallel. Such structure is slightly less pronounced for the samples with the highest filler content. *R*_q_ values for the oriented samples with the additives decreased when compared with that for the unoriented films, in general, which may result from better ordering of the chains in the oriented films. Probably during stretching the filler particles and the macromolecules adjusted to each other, which led to a greater ordering of macromolecules and the slight drop in *R*_q_ values of the samples after stretching. However, for the neat polymer, *R*_q_ value increased markedly after orientation due to the fibrillar structure. It was suggested that the fully developed fibrillar structure was achieved when the draw ratio was between 5 and 9 [26]. Thus, in this case the draw ratio was 3, which indicates partially fibrillar architecture.

The film morphology depends also on the stretching speed and drawing temperature. High stretching temperature and low stretching speed allow the macromolecules to disentangle and straighten otherwise the polymeric chains may break [26,33,34]. The studied films were oriented at elevated temperature and at high stretching speed, which promoted polymer photooxidation owing to the creating defects such as ruptures of polymer chains. These defects in the oriented samples caused a current breakdown during poling, which made the oriented samples with perlite and glass beads unsuitable for piezoelectric tests [6].

**Figure 8:**
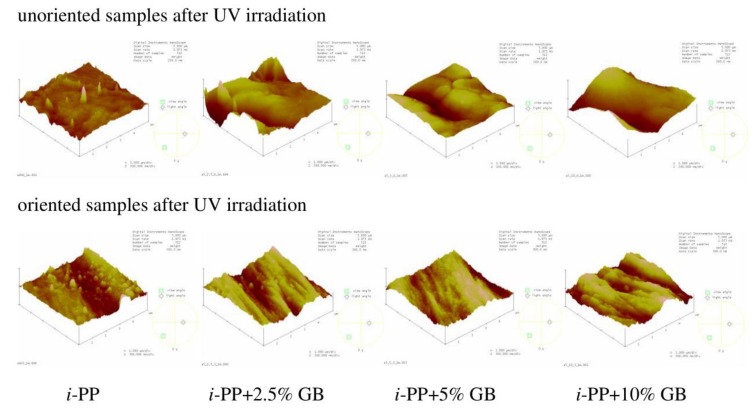
AFM images of UV irradiated unoriented (**top**) and oriented (**bottom**) neat *i*-PP film and *i*-PP films with different content of glass beads (GB). Scan area: 5 μm × 5 μm, z-scale for the unoriented films: 200 nm, for the oriented films: 300 nm.

After UV treatment the morphology of the unoriented neat *i*-PP film was still rough but with less amount of conical hills (Figure 8), *R*_q_ value increased only slightly probably due to the etching of amorphous phase less resistant to UV rays. The alterations in the surface morphology of the unoriented samples with the fillers were insignificant, *R*_q_ values changed irregularly. On the oriented neat *i*-PP film and the *i*-PP films with the fillers, one can notice small globular objects which may be composed of the low molecular weight products of the polymer photooxidation (Figure 8), *R*_q_ values changed only slightly for these films.

The effect of irradiation on morphology of the studied samples was not very clear, however, the photooxidation process could result in both formation of low molecular weight compounds that were able to fill in the hollows and the removal of amorphous phase from the film surface, which finally led to the irregular changes in *R*_q_ parameter.

## 4. Conclusions

The contact angle measurements, XPS and ATR-FTIR spectroscopy results showed that the studied *isotactic* polypropylene film had hydrophobic nature. The addition of the mineral fillers such as Sillikolloid, glass beads, or perlite to the *i*-PP film contributed to the production of more hydrophobic film surfaces as a result of some connections between hydrophilic groups of the fillers and polar groups of the polymer in which these groups existed as a contamination. The amount of the filler in the polymeric film was not as important as its type. Moreover, the orientation process led to a further reduction in polar groups at the surface, which might result from better adjustment of the film components during stretching at elevated temperature. Furthermore, the surface morphology and roughness of the polymeric films altered significantly when the mineral particles were added to the polymer and when the films were oriented. The orientation process led to completely different surface organization where partially fibrillar structures were formed.

UV irradiation of the samples resulted in the creation of new oxygen containing groups in the oriented and unoriented *i*-PP films with mineral fillers, which was detected by contact angle measurements, XPS and ATR-FTIR spectroscopy. These functional groups were formed owing to photooxidative degradation process. The presence of the additives and the orientation process facilitated polymer photooxidation. Both fillers and stretching created cavities in the polymer producing the cellular structure with trapped oxygen. The presence of voids in the polymer meant also an increase in the free volume and consequently greater mobility of intermediate photoproducts. The fillers contain iron oxide and the hydroxyl groups responsible for oxidation as well. Moreover, the oriented samples were more susceptible to oxidation than the unoriented ones because of some damages, e.g., chain cleavages and macroradicals formation that could occur during stretching. In these places the degradation processes start owing to the chromophoric groups already existing in the oriented films before UV action, while in the unoriented films the number of the defects was smaller.

The most effective photooxidation reactions occurred in the oriented samples with glass beads. These samples contained various amount of that filler (2.5–10%) but the efficiency of the process was similar. Moreover, the products of the polymer photooxidation were the same in all studied films suggesting that the presence of the fillers did not affect the mechanism of the polymer oxidation.

Longer UV irradiation of the studied samples in the artificial conditions resulted in significant surface oxidation of the polypropylene and also in its damage, which should be taken into account designing the piezomaterials. The presence of the fillers in the *i*-PP films resulted in the formation of the cellular architecture, which improved piezoelectric properties of *i*-PP films but deteriorated photostability of the modified polymer limiting potential applications of such materials outdoors or when they are exposed to UV radiation.

## Figures and Tables

**Table 1:** Fillers characteristics [5,6,16,17,18,19].

Filler	Producer	Chemical Analysis	Bulk Density	Particle Size	Particle Shape
Sillikolloid P87	Hoffman Mineral GmbH, Neuburg on the Danube, Germany	SiO_2_ (80%), Al_2_O_3_ (14%), Fe_2_O_3_ (<1%)	0.25 g/cm^3^	d97—6 μm, d50—1.5 μm	irregular round grains and lamellar stacks
Perlite, PEX-02/20	Mining and Metal Works—Zakłady Górniczo-Metalowe Zębiec, Poland	SiO_2_ (65–75%), Al_2_O_3_ (10–18%), K_2_O + Na_2_O (6–9%), MgO + CaO (2–6%), Fe_2_O_3_ (1–5%)	1–1.3 g/cm^3^	60% < 20 μm, 23.8%—20–32 μm, 5.4%—32–40 μm, 9.9%—40-63 μm, 0.6%—63–100 μm	the appearance of “crushed baubles”
Glass Beads MinTron 7™	Rock Tron, Bristol, United Kingdom	SiO_2_ (48–60%), Al_2_O_3_ (20–30%), Fe_2_O_3_ (3–7%), alkali oxides (5–9%)	~1.0 g/cm^3^	d90—20–30 μm, d50—5–9 μm	predominantly regular spherical shapes

Perlite and glass beads are amorphous but Sillikolloid contains crystalline phase [5,6].

**Table 2:** Surface free energy and its components for unoriented and oriented (O) isotactic-polypropylene (*i*-PP) films with different content of Sillikolloid, perlite, and glass beads (GB).

Sample	Surface Free Energy γ_s_ [mJ/m^2^]	Polar Component of Surface Free Energy γ_s_^p^ [mJ/m^2^]	Dispersion Component of Surface Free Energy γ_s_^d^ [mJ/m^2^]
*i*-PP	28.10	2.76	25.25
*i*-PP + 2.5% Sillikolloid	31.42	1.28	30.15
*i*-PP + 5% Sillikolloid	30.78	1.09	26.69
*i*-PP + 10% Sillikolloid	30.22	1.74	28.48
*i*-PP + 2.5% perlite	30.98	1.21	29.77
*i*-PP + 5% perlite	30.01	0.38	29.63
*i*-PP + 10% perlite	30.91	0.62	30.30
*i*-PP + 2.5% GB	31.16	0.44	30.72
*i*-PP + 5% GB	32.11	0.48	31.62
*i*-PP + 10% GB	32.37	0.10	32.27
*i*-PP O	30.30	2.62	27.68
*i*-PP O + 2.5% Sillikolloid	29.24	1.07	28.17
*i*-PP O + 5% Sillikolloid	34.25	0.50	33.75
*i*-PP O + 10% Sillikolloid	32.58	1.12	31.46
*i*-PP O + 2.5% perlite	32.62	0.16	32.46
*i*-PP O + 5% perlite	31.01	0.11	30.91
*i*-PP O + 10% perlite	32.77	0.09	32.68
*i*-PP O + 2.5% GB	33.57	0.20	33.37
*i*-PP O + 5% GB	34.42	0.24	34.18
*i*-PP O + 10% GB	36.58	0.22	36.36

**Table 3:** Surface free energy and its components for 1-month-irradiated unoriented and oriented (O) *isotactic* polypropylene (*i*-PP) films with different content of Sillikolloid, perlite, and glass beads (GB).

Sample	Surface Free Energy γ_s_ [mJ/m^2^]	Polar Component of Surface Free Energy γ_s_^p^ [mJ/m^2^]	Dispersion Component of Surface Free Energy γ_s_^d^ [mJ/m^2^]
*i*-PP	29.82	4.63	25.19
*i*-PP + 2.5% Sillikolloid	35.13	9.47	25.66
*i*-PP + 5% Sillikolloid	34.53	9.09	25.44
*i*-PP + 10% Sillikolloid	36.07	9.66	26.41
*i*-PP + 2.5% perlite	26.84	3.53	32.32
*i*-PP + 5% perlite	31.18	3.98	27.20
*i*-PP + 10% perlite	30.45	4.81	25.60
*i*-PP + 2.5% GB	36.44	9.60	26.84
*i*-PP + 5% GB	34.20	8.80	25.40
*i*-PP + 10% GB	40.82	4.77	36.06
*i*-PP O	35.06	5.07	30.0
*i*-PP O + 2.5% Sillikolloid	36.01	6.53	29.48
*i*-PP O + 5% Sillikolloid	37.03	2.73	34.30
*i*-PP O + 10% Sillikolloid	32.93	5.40	27.53
*i*-PP O + 2.5% perlite	37.00	3.86	33.14
*i*-PP O + 5% perlite	37.12	4.51	32.61
*i*-PP O + 10% perlite	35.41	6.05	29.36
*i*-PP O + 2.5% GB	38.76	10.50	28.26
*i*-PP O + 5% GB	41.91	12.78	29.13
*i*-PP O + 10% GB	38.80	10.65	28.15

**Table 4:** Surface concentration of elements calculated from X-ray photoelectron spectroscopy (XPS) for the unoriented and oriented (O) isotactic-polypropylene (*i*-PP) films with 5% of Sillikolloid, perlite, and glass beads (GB) for the samples before UV irradiation and after one month of irradiation.

Sample	Unirradiated			1 Month of UV Irradiation		
	C 1s (% at.)	O 1s (% at.)	Si 2p (% at.)	C 1s (% at.)	O 1s (% at.)	Si 2p (% at.)
*i*-PP	97	2	1	93	5	2
*i*-PP + 5% Sillikolloid	98	2	-	93	5	2
*i*-PP + 5% perlite	99	1	-	91	6	3
*i*-PP + 5% GB	97	3	-	96	4	-
*i*-PP O	99	1	-	96	4	-
*i*-PP O + 5% Sillikolloid	99	1	-	93	6	1
*i*-PP O + 5% perlite	100	0	-	95	5	-
*i*-PP O + 5% GB	99	1	-	94	6	-

**Table 5:** The values of the roughness parameter (*R*_q_, nm) for unoriented and oriented (O) *isotactic* polypropylene (*i*-PP) films with different content of Sillikolloid, perlite, glass beads (GB), before and after 1 month of UV irradiation. A scan area: 5 μm × 5 μm.

Sample	*R*_q_ [nm]		Sample	*R*_q_ [nm]	
	0 h	1 Month-Irradiated		0 h	1 Month-Irradiated
*i*-PP	13.8	17.4	*i*-PP O	32.1	30.0
*i*-PP + 2.5% Sillikolloid	44.9	45.5	*i*-PP O + 2.5% Sillikolloid	25.7	37.6
*i*-PP + 5% Sillikolloid	45.9	33.1	*i*-PP O + 5% Sillikolloid	20.0	41.0
*i*-PP + 10% Sillikolloid	76.0	56.7	*i*-PP O + 10% Sillikolloid	33.1	26.3
*i*-PP + 2.5% perlite	44.6	45.2	*i*-PP O + 2.5% perlite	32.7	47.3
*i*-PP + 5% perlite	29.5	42.1	*i*-PP O + 5% perlite	38.0	32.8
*i*-PP + 10% perlite	43.4	52.1	*i*-PP O + 10% perlite	50.6	62.8
*i*-PP + 2.5% GB	44.3	48.1	*i*-PP O + 2.5% GB	36.0	37.9
*i*-PP + 5% GB	42.9	23.6	*i*-PP O + 5% GB	36.4	44.7
*i*-PP + 10% GB	38.3	57.1	*i*-PP O + 10% GB	30.8	44.8
